# Low on-clopidogrel ADP- and TRAP-6-induced platelet aggregation in patients with atrial fibrillation undergoing percutaneous coronary intervention: an observational pilot study

**DOI:** 10.1007/s11239-023-02937-0

**Published:** 2024-02-12

**Authors:** Diona Gjermeni, Viktoria Anfang, Hannah Vetter, Sofia Szabó, David Hesselbarth, Nadine Gauchel, Patrick M. Siegel, Klaus Kaier, Alexander Kille, Kilian Franke, Stefan Leggewie, Dietmar Trenk, Daniel Duerschmied, Christoph Bode, Dirk Westermann, Christoph B. Olivier

**Affiliations:** 1 Department of Cardiology and Angiology, University Heart Center Freiburg - Bad Krozingen, Faculty of Medicine, University of Freiburg, Freiburg, Germany; 2 Department of Cardiology, Angiology, Haemostaseology and Medical Intensive Care, Medical Center Mannheim, Medical Faculty Mannheim, Heidelberg University, Heidelberg, Germany; 3 European Center for AngioScience (ECAS) and German Center for Cardiovascular Research (DZHK) Partner Site Heidelberg/Mannheim, Mannheim, Germany

**Keywords:** Atrial fibrillation, Percutaneous coronary intervention, Platelet aggregation, Dual anti-platelet therapy, Direct oral anticoagulants

## Abstract

High on-clopidogrel platelet reactivity (HPR) associates with ischemic risk in patients after percutaneous intervention (PCI). This study aimed to evaluate the association of HPR as assessed by multiple electrode aggregometry (MEA) with ischemic, thromboembolic, and bleeding risk in patients with atrial fibrillation (AF) undergoing PCI. Patients with AF and an indication for oral anticoagulation (OAC) were included in this prospective cohort study on day 1–3 after PCI. Platelet aggregation [U] was analyzed by MEA. HPR and low platelet reactivity (LPR) were defined as ADP-induced aggregation ≥ 46 U and ≤ 18 U, respectively. TRAP-6-induced aggregation reference was 94–156 U. The primary outcome was time to all-cause death, myocardial infarction, or stroke at 6 months. The secondary outcome was time to non-major clinically relevant bleedings or major bleedings. 159 patients were enrolled between May 2020 and May 2021. The median age was 78 years (interquartile range 72–82) and 111 (70%) were male. Median ADP- and TRAP-induced aggregation were 12 (6–17) and 49 (35–68) U, respectively. 147 (93%) patients had a low overall aggregability. HPR was detected in 2 patients (1%) and 125 (79%) had LPR. ADP-induced aggregation did not significantly associate with the primary outcome (r = 0.081, p = 0.309) but correlated inversely with bleeding risk (r = − 0.201, p = 0.011). HPR status as assessed by MEA among patients with AF after PCI was rare and overall aggregability was low. Conventional cut-off values for HPR might be inappropriate for these patients. ADP-induced aggregation might be helpful to identify patients at risk for bleeding.

**Supplementary Information:**

The online version contains supplementary material available at 10.1007/s11239-023-02937-0.

## Highlights

What is known about this topic?

- High on-clopidogrel platelet reactivity associates with ischemic risk in patients with dual antiplatelet therapy after percutaneous coronary intervention.
- In patients with atrial fibrillation, guidelines recommend oral anticoagulation and clopidogrel omitting acetylsalicylic acid after percutaneous coronary intervention.
- The association of platelet reactivity with outcomes is unknown in these patients at high risk for ischemic and bleeding events.
  What does the paper add?
- In this two-center study of patients with atrial fibrillation who receive oral anticoagulation and clopidogrel after percutaneous coronary intervention, high on-clopidogrel platelet reactivity as assessed by multiple electrode aggregometry was rare.
- Conventional cut-off values to detect high-on clopidogrel platelet reactivity might need to be re-evaluated in these patients to identify patients at increased ischemic risk.
- Thrombin receptor activating peptide 6-induced platelet aggregation was low in these patients indicating a reduced overall platelet aggregability.
- Low platelet reactivity indicates bleeding risk in patients with atrial fibrillation after percutaneous coronary intervention.

## Introduction

Dual antiplatelet therapy (DAPT) with acetylsalicylic acid (ASA) and a P2Y_12_ receptor antagonist reduces ischemic risk in patients with acute coronary syndrome (ACS) or chronic coronary syndrome (CCS) undergoing PCI [1, 2]. Atrial fibrillation (AF) is frequent in patients undergoing percutaneous coronary intervention (PCI) [1, 3]. For patients with non-valvular AF with an indication for oral anticoagulation (OAC), guidelines recommend short term triple antithrombotic therapy (TAT) after PCI followed by a dual regime with OAC and a P2Y_12_ receptor antagonist, omitting ASA [1, 2, 4]. Compared with vitamin K antagonists (VKA), direct oral anticoagulants (DOAC) are preferred OAC for most patients [4–10]. Compared with other P2Y_12_ Inhibitors, clopidogrel is the preferred choice for most patients due to lower bleeding risk [2, 11, 12].

Inter-patient variability of platelet inhibition has been observed in patients treated with clopidogrel after PCI. 15%–45% of the patients after PCI have high on-clopidogrel platelet reactivity (HPR) associated with higher risk for ischemic events [13–17]. Patients with AF when treated with OAC and clopidogrel and without ASA showing HPR after hospitalization might be at higher risk for ischemic events after PCI.

This study aimed to evaluate the association of HPR with ischemic, thromboembolic and bleeding risk in patients with AF undergoing PCI.

## Methods

### Study design and population

In this two-center prospective observational cohort pilot study, patients with AF undergoing PCI were enrolled between May 2020 and May 2021. The protocol was approved by the ethics committee of the Albert-Ludwigs-University Freiburg, Germany (registry number, 194/20). The study was registered to the German Clinical Trials Register (DRKS00021212). All patients had given their written consent before inclusion in the study. Patients with history of stent thrombosis, use of GPIIb/IIIa in the last 24 h or intake of another P2Y_12_ inhibitor than clopidogrel in the last 7 days were not suitable for enrolment (Table S1). Patients were grouped according to the presence of HPR.

### Coronary intervention

Patients presenting with ST-elevation myocardial infarction, non-ST-elevation myocardial infarction, unstable angina, or elective PCI were included. Arterial access was chosen at the discretion of the interventionalist. All patients received at the beginning 70–100 U/kg heparin for anticoagulation during the intervention. After the initial heparin doses further heparin administrations were adjusted according to the activated clotting time. All patients underwent coronary stenting with at least one drug-eluting stent (DES). All patients were treated with 75 mg clopidogrel and OAC after the procedure. TAT was prescribed at the discretion of the interventionalist. Discharge and management of in-hospital complications were performed per standard of care.

### Platelet aggregometry and blood samples

Venous blood was collected using a 21 G butterfly needle (Safety-Multifly®-Set, Sarstedt, Nümbrecht, Germany) and anticoagulated to a final concentration of  > 15 μg/ml r-hirudin (SARSTEDT Monovetten, Nümbrecht, Germany) on day 1–3 after PCI. Clopidogrel was administered orally and blood was drawn approximately 1 h later. Multiple electrode aggregometry (MEA, Roche Diagnostics, Rotkreuz, Switzerland) was performed within three hours after blood draw.

To assess the overall capacity of platelet aggregation, blood samples were stimulated with thrombin receptor activating peptide-6, TRAP-6 (final concentration 32 μM). Whole blood was stimulated with adenosine diphosphate, ADP (final concentration 6.4 μM) to monitor platelet reactivity. Aggregation was quantified as area under the curve (AUC) of aggregation units up to six minutes after addition of the stimulant. High on-clopidogrel platelet reactivity (HPR) was defined as ADP AUC ≥ 46 U (Units) and low platelet reactivity (LPR) as ADP AUC ≤ 18 U [14–16]. Reference values for normal TRAP-6-induced aggregation were defined as AUC 94–156 U as suggested by the manufacturer.

### Study outcomes and follow-up

The primary outcome was time to all-cause mortality, myocardial infarction, or stroke and was assessed at 6 months (± 2 weeks) [18]. The secondary outcome was time to non-major but clinically relevant bleedings (NMCR) or major bleedings according to International Society on Thrombosis and Haemostasis (ISTH) [19, 20].

Follow-up was performed by structured telephone interviews. If the structured telephone interview indicated an event, further documentation such as discharge letters, laboratory values, angiography reports, or death certificates were requested and the event package was presented to two independent physician reviewers blinded to MEA results. Major discrepancies were resolved by the principal investigator (CBO) who was blinded to MEA results.

### Statistical considerations

Categorical variables are presented as number and frequency and continuous variables as median with interquartile range (IQR). Mann–Whitney-U test was used to compare the median distribution of continuous variables. Pearson’s correlation coefficients were used for correlation analyses. Cox-regression was performed to analyze the number of patients free from ischemic, thromboembolic, and bleeding events until follow up. Receiver operating characteristic curves (ROC) were used for an estimation of the optimal aggregation cut-off values of our study population. To assess the association of variables with MEA multiple linear regression was used. Logistic regression was used to determine the association of variables with the clinical outcomes. All tests were 2-tailed and p-values ≤ 0.05 were considered statistically significant. Data were analyzed with Prism 9.2.0 (GraphPad Software, La Jolla, California, USA) and SPSS 27.0.0.1 (SPSS Inc, Chicago, Illinois, USA).

For the sample calculation we assumed an odds ratio (OR) of 5.1 for HPR status with primary composite ischemic outcomes compared with non-HPR status [21]. With an alpha threshold of 5%, 11 events provided 70% power to detect OR of 5.1. The power of 70% was chosen because of the exploratory nature of this study. With an event rate of 7.3% at 6 months [5], 151 patients were needed for this study. Assuming a 5% drop-out rate, 159 patients needed to be included in this study.

## Results

### Patient population and medication

From May 2020 to May 2021, 159 patients were included in this two-center observational prospective cohort study after PCI. One patient was excluded due to technical failure of MEA. The patient characteristics are provided in Table 1. Median age was 78 (IQR 72–82) years and 111 (70%) patients were male. Median CHA_2_DS_2_-VASc score was 5 (IQR 4–6) and median HAS BLED score was 3 (IQR 3–4). 45 (29%) patients had heart failure and 78 (49%) had a previous coronary intervention. 25% of the patients presented with an acute coronary syndrome (ACS).

**Table 1:** Baseline and procedural characteristics of the population

Baseline characteristics	Total n = 158
Male	111 (70%)
Age [years]	78 (72–82)
Body mass index [kg/m^2^]	27 (24–31)
CHA_2_DS_2_-VASC score	5 (4–6)
HAS BLED score	3 (3–4)
Type of atrial fibrillation	
Paroxysmal	93 (59%)
Persistent	31 (20%)
Permanent	34 (21%)
Laboratory data	
Creatinine [mg/dl]	1.1 (0.9–1.4)
Leucocytes [10^3^/µl]	7.9 (6.5–9.7)
Thrombocytes [10^3^/µl]	205 (174–267)
Hemoglobin [mg/dl]	12.8 (11.3–14)
Hematocrit [%]	38.2 (34–41.7)
IPF [%]	3.4 (2.4–4.9)
IPF absolute [10^3^/µl]	7.5 (5–10.3)
Medical history	
Transient ischemic attack or stroke	30 (19%)
Periphery artery vascular disease	23 (15%)
Heart failure	45 (29%)
Percutaneous coronary intervention	78 (49%)
Intracranial bleeding	5 (3%)
Gastrointestinal bleeding	8 (5%)
GFR (Cockroft-Gault) [ml/min]	70 (60–88)
Arterial hypertension	140 (89%)
Hyperlipidemia	125 (79%)
Diabetes mellitus	55 (35%)
Nicotine abuse	10 (6%)
Coronary artery disease	39 (25%)
Procedural characteristics	
Index event	
Elective	119 (76%)
Acute coronary syndrome	39 (25%)
Single vessel disease	54 (34%)
Left main disease	25 (16%)
Implanted Stents	
1	79 (50%)
> 1	79 (50%)

The values are n (%) or median (interquartile range, IQR). *IPF* Immature platelet fraction, *GFR* Glomerular filtration rate

Oral antithrombotic therapies at the time of procedure and at discharge are shown in Table 2. All patients had an indication for OAC. 131 (83%) received an ASA loading dose and 127 (80%) received clopidogrel loading dose with either 300 mg or 600 mg clopidogrel. 31 (20%) patients did not receive a clopidogrel loading dose; in these patients clopidogrel maintenance therapy had been established. 147 (93%) patients were treated with a combination therapy of ASA, clopidogrel, and OAC during hospitalization or longer. 11 (7%) patients received no ASA therapy. Baseline and procedural characteristics according to the ASA therapy regimen are shown in Table S3, supplement. All patients were discharged with clopidogrel. At discharge 155 (98%) patients were prescribed an OAC: 53 (34%) apixaban, 6 (4%) dabigatran, 18 (11%) edoxaban, 74 (47%) rivaroxaban, and 4 (3%) VKA (Table 2). 37 (23%) received ASA beyond hospitalization.

**Table 2:** Periprocedural medication and medication at discharge

Antithrombotic medication		Total n = 158
(Peri-)procedural medication		
ASA		
No ASA	11	(7%)
ASA maintenance therapy	14	(9%)
ASA Loading	131	(83%)
ASA beyond discharge only	2	(1%)
Clopidogrel		
Clopidogrel maintenance therapy	31	(20%)
Clopidogrel loading	127	(80%)
Prescribed medication at discharge		
ASA	37	(23%)
≤ 7 days	16	(10%)
8–30 days	15	(10%)
> 30 days	6	(4%)
Clopidogrel	158	(100%)
6 months	65	(41%)
> 6 months	92	(59%)
Oral anticoagulation	155	(98%)
Apixaban	53	(34%)
Dabigatran	6	(4%)
Edoxaban	18	(11%)
Rivaroxaban	74	(47%)
Vitamin-K-antagonist	4	(3%)

The values are n (%). *ASA* acetylsalicylic acid

Oral anticoagulation was paused prior to PCI in 146 (92%) of the patients. 14 (9%) patients were bridged with low weight molecular heparin (LWMH) or unfractionated heparin (UFH). Most of the patients were pre-treated with an OAC and only 9 (6%) were OAC-naïve.

### Platelet reactivity and association with the outcomes

**Fig. 1:**
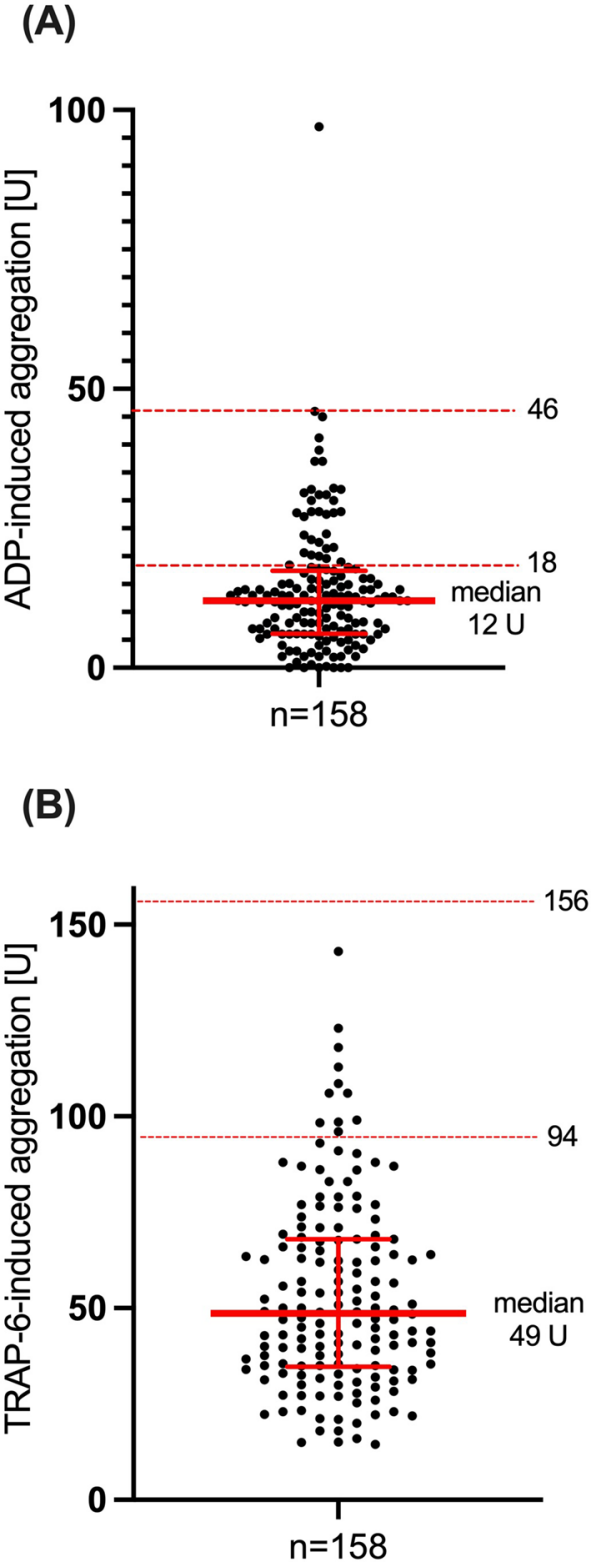
ADP (**A**)- and TRAP (**B**)- induced aggregation with median and interquartile range. Horizontal lines indicate conventional cut-off values for platelet reactivity (high or low, respectively) (**A**) or reference values (**B**), respectively

MEA was performed up to four days after PCI. Median ADP-induced aggregation was 12 (IQR 6–17) U (Fig. 1). 2 of 158 patients (1%) had HPR and 125 (79%) had LPR. Median TRAP-6-induced aggregation was 49 (IQR 35–68) U. 147 (93%) patients had a low overall aggregability. 11 (7%) patients had a TRAP-6-induced aggregation within reference limits. TRAP-6- and ADP-induced aggregation correlated significantly, (r = 0.580, p =  < 0.0001, Figure S2, supplement).

The primary outcome of all cause death, myocardial infarction, or stroke occurred in 11 (7%) patients (Table 3). None of these patients had HPR. Thus, association of HPR with the primary outcome was not calculated. The rates of myocardial infarction and stroke were low: 2 (1%) and 2 (1%) patients, respectively. Bleeding occurred in 57 (38%) patients. The majority of these bleedings were minor. The composite secondary outcome of major or non-major clinically relevant bleedings occurred in 23 (15%) patients. While the majority (9 of 11 [81%]) of the first ischemic and thromboembolic events were observed during the first 90 days after PCI, the secondary outcome of bleeding was equally distributed over the 6 months of follow-up (Figure S1, supplement).

**Table 3:** Primary and secondary outcomes at 6 months ± 2 weeks follow-up

Outcomes	Total n = 158	
Death, Stroke, or MI (primary outcome)	11	(7%)
Death	7	(4%)
Cardiovascular	2	(1%)
Non-cardiovascular	4	(3%)
Undetermined	1	(0.6%)
Myocardial infarction	2	(1%)
Stroke	2	(1%)
Hemorrhagic	1	(0.5%)
Undetermined	1	(0.5%)
Secondary outcomes		
NMCR or major (ISTH)	23	(15%)
NMCR	9	(6%)
Major	14	(9%)
Any bleeding	57	(36%)
Minor (ISTH)	34	(22%)
BARC type 3 or 5	13	(8%)

The values are in number and percentage, n (%).*MI* myocardial infarction, *ISTH* International Society on Thrombosis and Haemostasis, *BARC* Bleeding Academic Research Consortium

Neither ADP- nor TRAP-6 -induced aggregation was associated with the primary composite ischemic outcome. All ischemic/thromboembolic events occurred in patients taking ASA for the first time and no events occurred in patients that were taking ASA as maintenance therapy, p = 0.600. TRAP-6-induced aggregation formally associated with stroke (r = 0.166, p = 0.037). However, only two patients experienced strokes and one of these was hemorrhagic.

ADP-induced aggregation significantly associated with bleeding (r = − 0.201, p = 0.011; Table 4). There was a trend for TRAP-6-induced aggregation to associate with major bleedings (r = − 0.153, p = 0.055).

**Table 4:** Correlation of multiple electrode aggregometry with primary and secondary outcomes

Outcomes	ADP AUC [U]		TRAP AUC [U]	
	Correlation	p-value coefficient	Correlation	p-value coefficient
Death, stroke, and myocardial infarction	0.081	0.309	0.119	0.119
Death	0.063	0.430	0.083	0.302
CV- death	0.100	0.213	0.098	0.220
Myocardial infarction	− 0.530	0.505	− 0.046	0.563
Stroke	0.122	0.125	0.166	0.037
NMCR or major bleeding (ISTH)	− 0.108	0.176	− 0.115	0.152
NMCR bleeding	− 0.030	0.708	0.013	0.867
Major bleeding	− 0.110	0.169	− 0.153	0.055
Any bleeding	− 0.201	0.011	− 0.058	0.470
Minor bleeding (ISTH)	− 0.142	0.076	0.031	0.702
BARC type 3 or 5 bleeding	− 0.093	0.248	− 0.123	0.120

*CV* cardiovascular, *NMCR* non-major clinically relevant, *BARC* Bleeding Academic Research Consortium, *ISTH* International Society on Thrombosis and Haemostasis, *TRAP* thrombin receptor activating peptide-6, TRAP-6; ADP-adenosine diphosphate

Similar results were found when patients were grouped according to the presence or type of bleeding (Figure S3, supplement): median ADP-induced aggregation was significantly lower in patients who experienced bleeding during follow-up compared with those who did not (9 [IQR 5–14] vs 13 [IQR 7–20] U; p = 0.008). Median TRAP-6-induced aggregation was significantly lower in patients who experienced major bleeding compared with those who did not (36 [IQR 27–59] vs 49 [IQR 35–68] U; p = 0.045).

The ROC-AUC determined that both ADP- and TRAP-6-induced aggregation have a poor ability to predict ischemic, thromboembolic, and bleeding events in patients with AF undergoing PCI (Table 5). In a multivariable regression analysis, HAS-BLED score tended to be associated with the risk of NMCR or major bleedings (OR 1.91 [CI 0.96–3.79; p = 0.064], Table S4, supplement).

**Table 5:** Receiver operating characteristic area under the curve of TRAP- and ADP- induced aggregation for outcomes

MEA	AUC	Confidence interval	p-value
Primary outcome			
ADP AUC [U]	0.566	0.386–0.745	0.467
TRAP AUC [U]	0.641	0.455–0.826	0.120
Secondary outcome			
ADP AUC [U]	0.596	0.481–0.711	0.141
TRAP AUC [U]	0.580	0.456–0.703	0.223

The values are in number, n. *MEA* multiple electrode aggregometry

### Factors associated with platelet reactivity

149 (94%) patients were pre-treated with an OAC. ADP-induced aggregation values between patients pre-treated with an OAC and naive patients were similar. Median TRAP-6-induced aggregation was significantly higher in patients who had been recently started on OAC compared with the patients who were chronically treated with OAC (63 [IQR 52–77] vs 47 [IQR 34–68] U; p = 0.037), (Figure S4, supplement). Median of ADP- and TRAP-6-induced aggregation were similar across types of OAC (Figure S5, supplement).

Median ADP-induced aggregation was similar between patients who received clopidogrel loading and patients who received clopidogrel maintenance therapy, (Figure S6, supplement). There was no significant difference for ADP-induced aggregation when the doses of clopidogrel used for the loading was 300 mg or 600 mg, (Figure S6, supplement). The was no significant difference between the aggregation median values of ADP and TRAP-6 for patients that were first-time users of ASA and patients taking ASA as maintenance therapy, (Figure S7, supplement).

Time from clopidogrel loading to MEA did not correlate significantly with ADP-induced aggregation (Figure S8, supplement).

The majority (143/158 [90%]) of blood samples were analyzed within 2 h after draw. Median of ADP- and TRAP-6-induced aggregation were similar when MEA measurement was performed < 120 min (2 h) and > 120 min (between 2–3 h after blood draw), (Figure S9, supplement).

Supplementary Table S3 provides information on platelet aggregation according to the regimen of antiplatelet therapy.

A multivariable linear regression analysis was performed to assess the association of baseline characteristics, laboratory parameters and periprocedural medication with platelet aggregation (Table S2, supplement). Platelet count associated with higher ADP-induced aggregation. Enrolling site did not significantly associate with aggregation values. Distribution of ADP-, TRAP-6- and arachidonic acid (AA)-induced aggregation values according to the site where the measurement was performed is shown in Figure S10, supplement. ACS at time of presentation was not significantly associated with ADP-induced aggregation (12 [IQR 7–17] U patients without ACS vs 8 [IQR 4–18] U patients presenting with ACS; p = 0.091).

## Discussion

The findings of this observational study of patients with AF undergoing PCI were that (1) HPR_ADP_ status as assessed by MEA was very low with only 1%, (2) overall platelet aggregability as assessed by TRAP-6-induced aggregation was reduced in this patient cohort, and (3) low aggregation values indicated higher bleeding risk.

In patients with DAPT therapy undergoing PCI (without AF) higher rates of high on-clopidogrel therapy were described compared with this observational study [15–17]: The TROPICAL-ACS trial included patients with ACS. Platelet function testing (PFT) was assessed with MEA and HPR rates were 39% [17]. Platelet aggregation was assessed with VerifyNow after PCI in 8000 patients and 35% of the patients showed HPR when treated with DAPT (clopidogrel and ASA) [16].

Very few studies specifically evaluated the platelet aggregation in patients treated with antiplatelet therapy and OAC. One observational study showed 15% HPR in patients treated with triple therapy consisting of DAPT and VKA [22]. VKA might attenuate the effect of clopidogrel on platelet aggregation [23, 24] and higher ADP- induced aggregation values were shown in a study when compared with DOAC [25]. This might explain the higher HPR rates in patients with TAT treated with a VKA compared with the present study [22, 23] and there is a consistency with other studies suggesting lower ADP-included aggregation when treated with DOAC [25].

Platelet function may be altered if DOAC is used for a longer period probably due to a change in the expression profile of thrombin receptor especially under therapy with dabigatran [26, 27]. 94% of patients included in the present study were pre-treated with a DOAC but only, few (4%) of the patients were treated with dabigatran.

Median ADP-induced aggregation was similar when OAC was given for longer time compared to when OAC was recently started.

In patients treated with edoxaban in combination with DAPT, TRAP-6 -induced aggregation increased following ASA withdrawal, particularly with low-dose edoxaban [28]. In this study, TRAP-6-induced aggregation was mostly below the reference values. If the patients were treated for longer time with OAC, median TRAP-6-induced aggregation was significantly lower compared with recently initiated OAC therapy, (Figure S4, supplement). TRAP-6-induced aggregation was increased significantly if OAC was interrupted for more than 4 days. However, in a multivariable regression, the pause of OAC was not associated with TRAP-6-induced aggregation.

The primary composite ischemic outcome occurred in 11 (7%) patients whereas the secondary outcome consisting of NMCR and major bleedings (ISTH) [19, 20] occurred in 23 (15%) patients similar to other studies [4–10]. The incidence of bleeding events was constant during the 6 months of follow-up but most of major adverse cardiovascular events (MACE) occurred in the first 90 days of follow-up in this study. This finding is consistent with other trials comparing DOAC therapy with apixaban [5], edoxaban [6] and dabigatran [7] with VKA therapy in patients undergoing PCI and AF.

Platelet aggregation associated with ischemic and bleeding risk, especially in a setting of patients treated with DAPT after PCI [13–16]. In a meta-analysis, HPR associated with a 2–fourfold increased risk for stent thrombosis and in contrast low platelet reactivity (LPR) with higher bleeding risk (relative risk, RR 1.74 [CI 1.47–2.06]; p =  < 0.01) [15]. A recent study observed no association between HPR and MACE in patients with AF undergoing PCI with TAT [23]. Neither TRAP-6- nor ADP-induced aggregation were a strong predictor of both ischemic and bleeding risk in this study. Nevertheless, the upper value of the 95% confidence interval (CI) for AUC of ADP-induced aggregation was 0.745, leading to uncertain conclusions regarding the association of platelet aggregation as assessed by MEA with the primary composite ischemic outcome. This study identified a formal association of TRAP-6 -induced aggregation with stroke. However, only two patients experienced strokes and one of these was haemorrhagic.

This study identified ADP-induced aggregation as a marker of bleeding risk: ADP-induced aggregation correlated significantly with the total bleedings and median ADP- induced aggregation was numerically lower in patients with major bleeding vs no bleeding. This finding is consistent with other studies suggesting that patients undergoing PCI are at higher risk for bleeding events [15]. HPR status related inversely with bleedings in patients undergoing PCI [16].

TRAP-6-induced aggregation correlated with major bleeding risk inversely. Since TRAP-6-induced aggregation represents overall aggregability, it is biologically plausible that lower values relate to a higher bleeding risk. Thus, platelet function testing might be useful to guide antiplatelet therapy, such as shortening duration of antiplatelet therapy following PCI in patients on OAC due to AF. The definition of HPR according to ADP-induced aggregation as assessed by MEA are provided by a consensus document [14]. This study described a low rate of patients with HPR and a reduced overall platelet aggregability. The reason of this reduced platelet aggregation in these patients is unknown but was consistent across two independent sites. Thus, the validity of these cut-off values might need to be re-evaluated in this specific population at high bleeding and ischemic risk. However, rates of HPR may depend on the method used to measure platelet reactivity [29]. The association of platelet reactivity as assessed by MEA in patients with clopidogrel and OAC should be explored in further studies.

### Limitations of the study

The low rate of HPR and the small population size limit the ability to identify less pronounced associations of MEA with the outcomes. Further assays to gain insights on interaction of DOAC with platelets to gain with potential influence on the findings, such as interaction of DO have not been assessed. This study was an observational pilot study and should be regarded as hypothesis generating. The two-center character of this study limits the generalizability of the results. Other pre-analytic factors such as time of measurement after blood draw might have impacted the aggregation values [30]. Recent study showed that aggregation values did not differ according to the time of measurement after blood draw [31] in patients with coronary artery syndrome.

Although interventionalists who prescribed concomitant antithrombotic therapy were blinded to MEA results, known factors associated with HPR might have influenced therapy choices associated with outcomes. A study suggested that status of clopidogrel platelet reactivity may vary in time after PCI [32]. Thus, a limitation of this study is the platelet function assessment at a single time point in stead of serial measurements. No measurements in healthy volunteers were performed for comparison.

## Conclusion

In this observational study, HPR status as assessed by MEA among patients with AF after PCI was rare. Low TRAP-6-induced aggregation indicated a reduced overall aggregability as assessed by MEA in these patients. Conventional cut-off values to detect high-on clopidogrel platelet reactivity might need to be re-evaluated in patients with AF and OAC following PCI. Given the low rate of HPR, this study was underpowered to detect a significant association with ischemic and thromboembolic outcomes and the results are inconclusive. However, reduced platelet aggregation might be helpful to identify patients with atrial fibrillation at risk for bleeding to guide antithrombotic therapy after PCI.

### Supplementary Information

Below is the link to the electronic supplementary material.

Supplementary file1 (DOCX 2575 kb)
