# Supplementary material for: Low on-clopidogrel ADP- and TRAP-6-induced platelet aggregation in patients with atrial fibrillation undergoing percutaneous coronary intervention: an observational pilot study
Source: J Thromb Thrombolysis. 2024 Feb 12;57(3):361–9. doi: 10.1007/s11239-023-02937-0 (PMC10961278; doi:10.1007/s11239-023-02937-0)
Supplement: Supplementary file 1 — Supplementary file1 (DOCX 2575 kb) [file 11239_2023_2937_MOESM1_ESM.docx]

# SUPPLEMENT

## Table S1. Key inclusion and exclusion criteria

| **Inclusion criteria** |
| --- |
| - Informed consent - ≥18 years of age or older - Atrial fibrillation with an indication for oral anticoagulation (i.e. CHA2DS2VASC score ≥1 for males, ≥2 for females) - Percutaneous coronary intervention in the first 3 days |
| **Exclusion criteria** |
| - Contraindication to a DOAC (i.e. apixaban, dabigatran, edoxaban, rivaroxaban) or clopidogrel - History of stent-thrombosis - Uncompliant patient from the point of view of the principal investigator like for example.: assumed non-compliance, frequent use of alcohol and drugs or not willing to proceed according to the protocol (patient not willing to follow-up) - GPIIb/IIa inhibitor in the last 24h - Use of prasugrel or ticagrelor in the last 7 days |

## Table S2. Multiple linear regression analysis evaluating the impact of baseline characteristics, laboratory values and periprocedural medication on TRAP-6-, ADP- and AA- induced aggregation

|  | **TRAP AUC [U]** | | **ADP AUC [U]** | | **AA AUC [U]** | |
| --- | --- | --- | --- | --- | --- | --- |
| **Independent variables** | **ß-coefficient (CI95%)** | **p-value** | **ß-coefficient (CI95%)** | **p-value** | **ß-coefficient (CI95%)** | **p-value** |
| Inclusion center (Freiburg/ Bad-Krozingen) | -0.063 (-11.113-4.865) | 0.441 | -0.078 (-5.316-2.239) | 0.422 | 0.102 (-3.070-10.976 | 0.268 |
| Male sex | 0.025 (-6.460-9.133) | 0.735 | 0.070 (-2.207-5.166) | 0.429 | -0.113 (-11.610-2.098) | 0.172 |
| Age | -0.049 (-0.602-0.296) | 0.501 | -0.660 (-2.92-.132) | 0.457 | -0.049 (-.512-.277) | 0.556 |
| Body mass Index (kg/m2) | 0.083 (-0.344-1.195) | 0.276 | 0.135 (-.092-.636) | 0.142 | -0.093 (-1.044-.309) | 0.284 |
| Heart failure | 0.073 (-3.838-11.919) | 0.312 | 0.120 (-1.113-6.338) | 0.168 | 0.066 (-4.087-9.766) | 0.419 |
| Diabetes mellitus | -0.044 (-9.940-5.368) | 0.556 | 0.027 (-3.053-4.184) | 0.758 | 0.013 (-6.214-7.244) | 0.880 |
| Thrombocytes [10^3^/µl] | 0.589 (0.121-0.199) | <0.001 | 0.184 (.001-.038) | 0.036 | 0.387 (.048-.116) | <.001 |
| Leucocytes [10^3^/µl] | -0.004 (-0.555-0.527) | 0.958 | -0.065 (-.355-.157) | 0.444 | 0.110 (-.145-.806) | 0.171 |
| Acute coronary syndrome | 0.056 (-5.383-11.843) | 0.460 | -0.106 (-6.470-1.676 | 0.246 | -0.048 (-9.732-5.411) | 0.573 |
| Doses of clopidogrel loading (mg) | 0.071 (-0.007-0.022) | 0.331 | -0.123 (-.012-.002) | 0.162 | -0.040 (-.016-.010) | 0.628 |
| Doses of ASA-loading (mg) | -0.093 (-0.040-0.009) | 0.210 | -0.002 (-.12-.011) | 0.982 | -0.178 (.045--.002) | 0.340 |
| Pause of oral anticoagulation when performing MEA (yes/no) | -0.002 (-10.116-9.848) | 0.979 | -0.003 (-4.801-4.640) | 0.973 | 0.007 (-8.425-9.126) | 0.937 |
| Number of days of pause of OAC before MEA | -0.044 (-1.863-1.782) | 0.965 | -0.005 (-.883-.841) | 0.962 | -0.021 (-1.792-1.412) | 0.815 |

Abbreviations: Beta, standardized regression coefficient; CI, confidence intervals; ASA, acetylsalicylic acid; OAC, oral anticoagulation; MEA, multiple electrode aggregometry.

## Table S3. Baseline and procedural characteristics according to the antiplatelet therapy regimen

| **Characteristic** | **Total**  **N=158** | | **ASA Therapy** | | | | | |  |
| --- | --- | --- | --- | --- | --- | --- | --- | --- | --- |
|  |  |  | **No ASA**  **N=11** | | **During hospitalization only**  **N=110** | | **Beyond discharge**  **N=37** | | **p-value** |
| Demographics |  |  |  |  |  |  |  |  |  |
| Age [years] | 78 | (72-82) | 76 | (67-80) | 79 | (72-82) | 78 | (72-81) | 0.487 |
| Male | 111 | (70%) | 9 | (82%) | 75 | (68%) | 27 | (73%) | 0.588 |
| Medical History |  |  |  |  |  |  |  |  |  |
| CHA_2_DS_2_-VASc score | 5 | (4-6) | 4 | (4-6) | 5 | (4-6) | 5 | (4-6) | 0.880 |
| HAS-BLED | 3 | (3-4) | 4 | (2-4) | 3 | (3-4) | 3 | (3-4) | 0.692 |
| Heart failure | 45 | (29%) | 3 | (27%) | 30 | (27%) | 12 | (33%) | 0.831 |
| Arterial hypertension | 140 | (89%) | 9 | (82%) | 97 | (88%) | 34 | (92%) | 0.632 |
| Diabetes Mellitus | 55 | (35%) | 5 | (46%) | 37 | (34%) | 13 | (35%) | 0.734 |
| GI-Bleeding | 8 | (5%) | 0 | (0%) | 3 | (3%) | 5 | (14%) | 0.026 |
| Intracranial Bleeding | 5 | (3%) | 1 | (9%) | 3 | (3%) | 1 | (3%) | 0.508 |
| PAD | 23 | (15%) | 2 | (18%) | 15 | (14%) | 6 | (16%) | 0.876 |
| GFR [ml/min] | 70 | (60-88) | 69 | (60-91) | 69 | (60-87) | 75 | (64-92) | 0.548 |
| TIA/Stroke | 31 | (20%) | 2 | (18%) | 20 | (18%) | 9 | (24%) | 0.713 |
| MI | 31 | (20%) | 3 | (27%) | 20 | (18%) | 8 | (22%) | 0.724 |
| Previous PCI | 78 | (50%) | 8 | (73%) | 49 | (45%) | 21 | (57%) | 0.120 |
| Hyperlipidemia | 125 | (79%) | 11 | (100%) | 88 | (80%) | 26 | (70%) | 0.095 |
| Procedural Characteristics |  |  |  |  |  |  |  |  |  |
| One-Vessel CAD | 54 | (43%) | 6 | (55%) | 38 | (35%) | 10 | (27%) | 0.450 |
| Left Main Disease | 25 | (16%) | 2 | (18%) | 17 | (16%) | 6 | (16%) | 0.970 |
| Number of Stents |  |  |  |  |  |  |  |  | 0.237 |
| 1 | 54 | (34%) | 6 | (54%) | 38 | (34%) | 10 | (27%) |  |
| >1 | 104 | (66%) | 5 | (46%) | 72 | (66%) | 27 | (73%) |  |
| Index Event of PCI |  |  |  |  |  |  |  |  | 0.827 |
| Elective | 119 | (75%) | 10 | (91%) | 81 | (74%) | 28 | (76%) |  |
| Acute | 39 | (25%) | 1 | (9%) | 29 | (26%) | 9 | (24%) |  |
| NOAC at discharge | 155 | (98%) | 11 | (100%) | 110 | (100%) | 35 | (95%) | 0.036 |
| Platelet Aggregation AUC [U] |  |  |  |  |  |  |  |  |  |
| TRAP-induced | 49 | (35-68) | 41 | (25-56) | 49 | (35-66) | 48 | (36-77) | 0.472 |
| ADP-induced | 12 | (6-17) | 13 | (8-23) | 12 | (6-16) | 13 | (7-17) | 0.645 |
| The values are number and percentage, n (%) or median (interquartile range [IQR]). Abbreviations: ASA, acetylsalicylic acid; GI-Bleeding, gastrointestinal Bleeding; MI, myocardial infarction; NOAC, non-Vitamin K antagonistic oral anticoagulation; PCI, percutaneous coronary; intervention; TIA, transient ischemic attack, CAD, coronary artery disease; PAD, peripheral arterial occlusive disease; AUC, area under the curve; TRAP, Thrombin receptor activating peptide; ADP, adenosine diphosphate; AA, arachidonic Acid; U, units. | | | | | | | | | |

## Table S4. Association of baseline characteristics with primary ischemic outcome, and secondary bleeding outcome

|  | **Death, stroke, MI** | | | | | **NMCR + major bleeding** | | |
| --- | --- | --- | --- | --- | --- | --- | --- | --- |
| **Characteristic** | **Odds Ratio** | **95% CI** | **p-value** | | **Odds**  **Ratio** | | **95% CI** | **p-value** |
| **Baseline characteristics** |  |  |  | |  | |  |  |
| Gender male | 1.103 | 0.152; 7.999 | 0.923 | | 2.210 | | 0.493; 9.916 | 0.300 |
| Age | 1.116 | 0.929; 1.341 | 0.240 | | 1.170 | | 1.014; 1.352 | 0.032 |
| Body mass Index (kg/m^2^) | 0.871 | 0.601; 1.264 | 0.468 | | 0.807 | | 0.618; 1.053 | 0.114 |
| CHA_2_DS_2_-VASC score | 0.924 | 0.469; 1.818 | 0.818 | | 0.680 | | 0.396; 1.165 | 0.160 |
| HASBLED score | 1.478 | 0.589; 3.707 | 0.405 | | 1.910 | | 0.962; 3.792 | 0.064 |
| **Medical history** |  | | |  | | | | |
| Heart failure | 1.215 | 0.225; 6.566 | 0.821 | | 1.822 | | 0.580; 5.725 | 0.304 |
| Type of AF | 0.404 | 0.127; 1.291 | 0.126 | | 1.123 | | 0.603; 2.094 | 0.715 |
| Myocardial infarction | 1.098 | 0.231; 5.222 | 0.906 | | 0.651 | | 0.176; 2.413 | 0.521 |
| Intracranial bleeding | 7.245 | 0.371; 141.464 | 0.192 | | 0.924 | | 0.031; 28.009 | 0.964 |
| Gastrointestinal bleeding | 0.694 | 0.028; 16.961 | 0.823 | | 6.461 | | 1.115; 37.434 | 0.037 |
| GFR | 1.024 | 0.909; 1.154 | 0.693 | | 1.054 | | 0.969; 1.146 | 0.221 |
| Diabetes | 1.091 | 0.213; 5.576 | 0.917 | | 1.552 | | 0.481; 5.015 | 0.462 |
| Arterial hypertension | 1.323 | 0.095; 18.394 | 0.835 | | 2.456 | | 0.202; 29.924 | 0.481 |
| Abbreviations: AF, atrial fibrillation; CI, confidence interval; GFR, glomerular filtration rate; MACE, major adverse cardiovascular events; NMCR, non-major clinically relevant bleedings. | | | | | | | | |

## Figure S1. Kaplan-Meier-curves of the primary ischemic outcome (A) and bleeding outcome (B) until follow-up at 180 days (6 months)

**
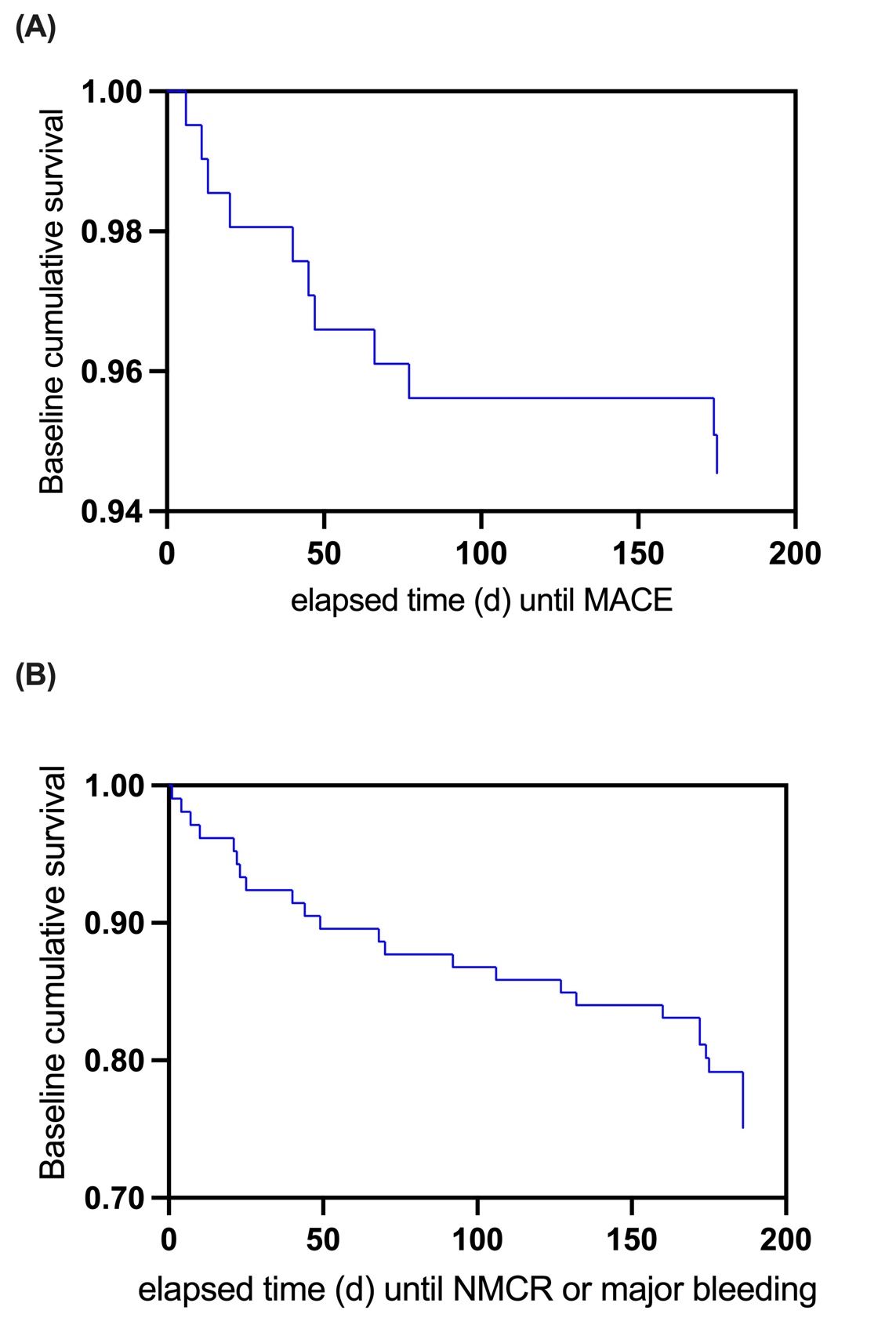
**

## Figure S2. Correlation of ADP- and TRAP- induced aggregation.


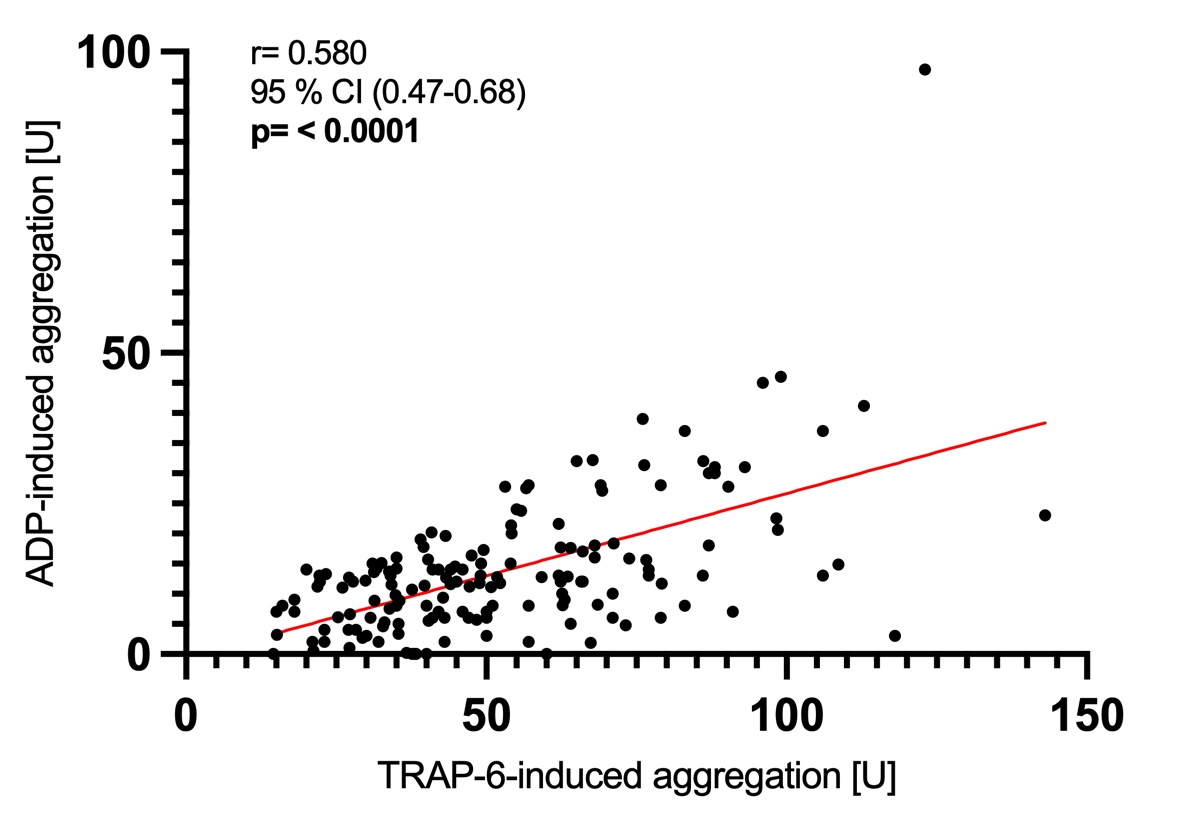


## Figure S3. Median distribution according to the presence or the type of bleeding complications for (A) ADP and (B) TRAP-induced aggregation

**
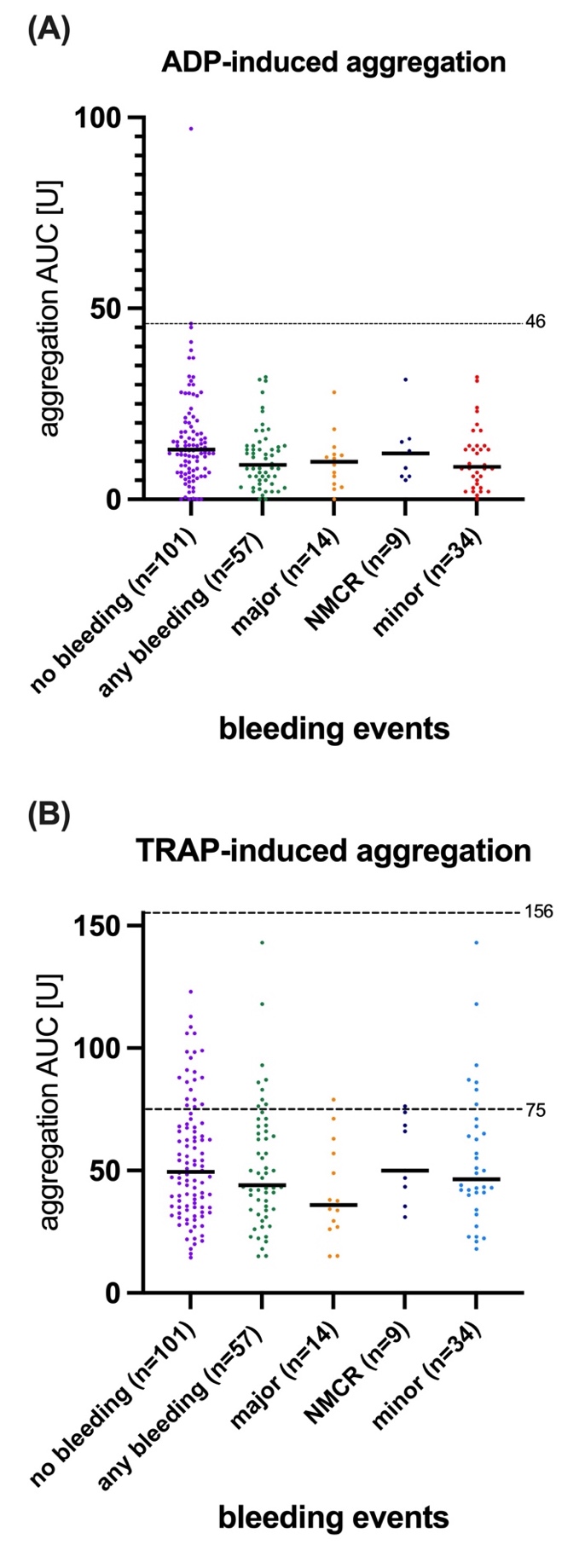
**

Interrupted line (46 U) in figure A represents the suggested cut-off for defining high platelet reactivity for ADP-induced aggregation whereas the two interrupted lines in figure B (94U and 156U) represent the lower and higher reference value for TRAP- induced aggregation respectively. Black line represents median of ADP and TRAP-induced aggregation values for each subgroup.

## Figure S4. (A) ADP and (B) TRAP- induced aggregation in patients on long-term OAC therapy compared with patients on newly started OAC

**
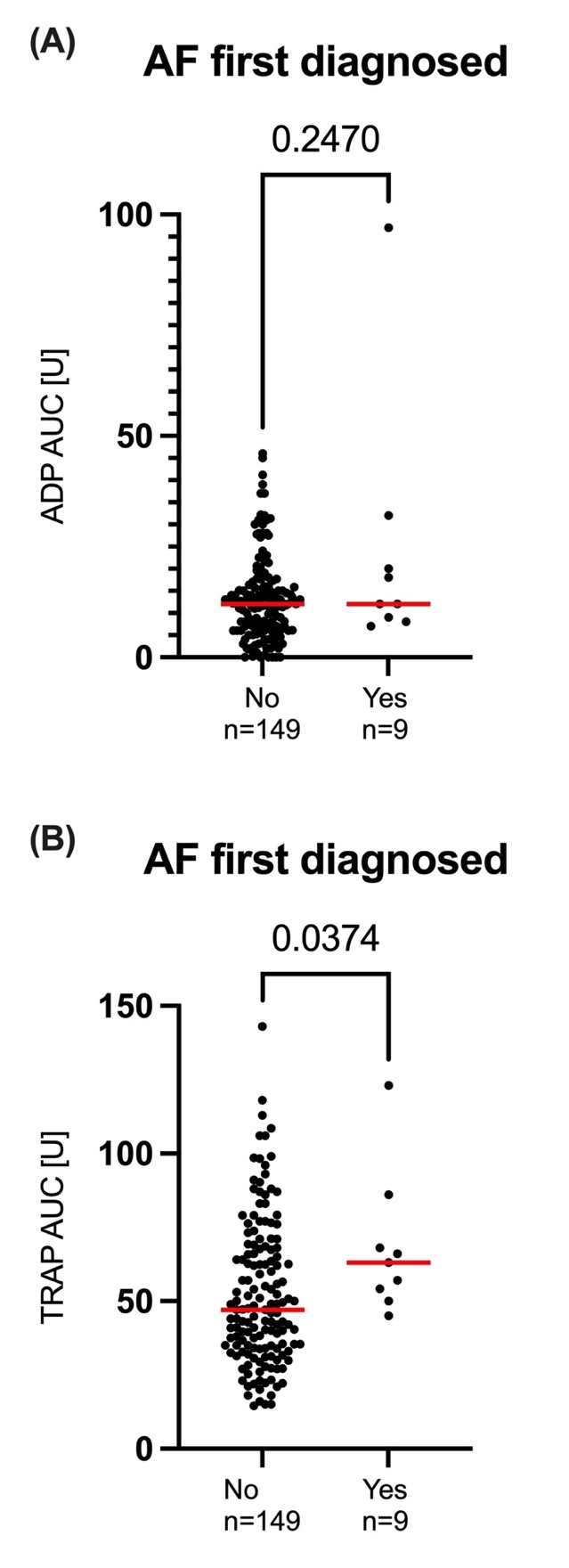
**

Red line shows median for ADP and TRAP- induced aggregation values measured for each of the groups.

## Figure S5. (A) ADP and (B) TRAP- induced aggregation and type of oral anticoagulation used


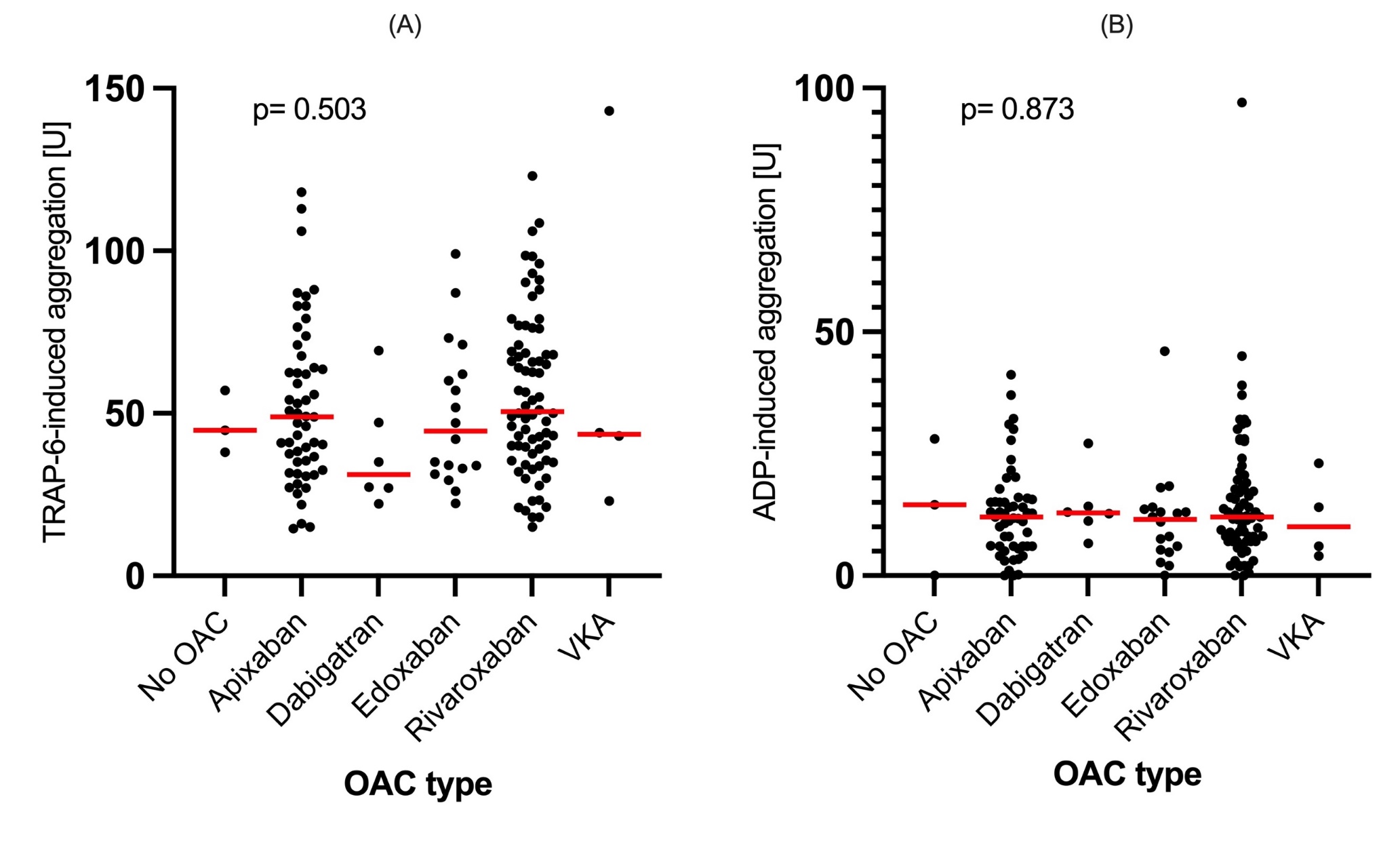


## Figure S6. ADP-induced aggregation according to the (A) performance of loading with clopidogrel or (B) doses of loading

**(A)
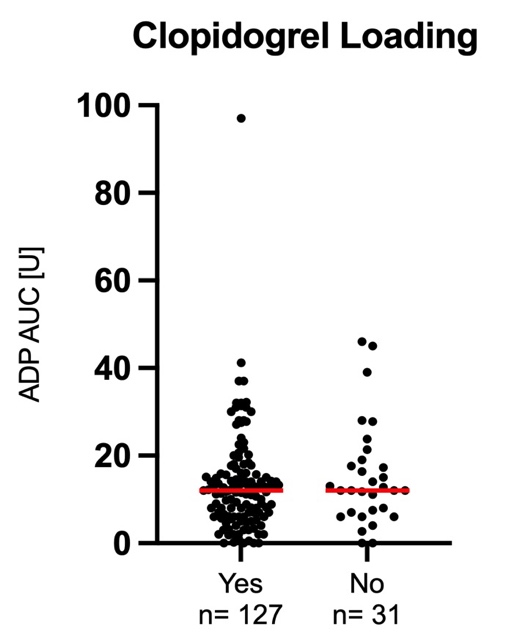
**

**(B)
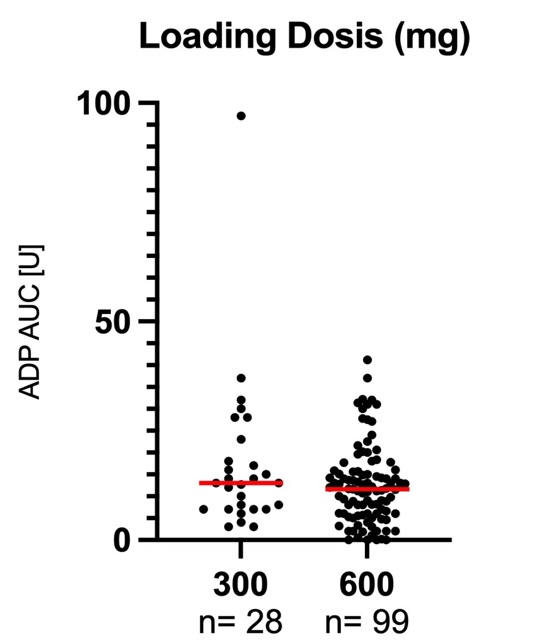
**

Red line shows median for ADP- induced aggregation values measured for each of the groups.

**Figure S7. (A)TRAP- and ADP- induced aggregation for acetylsalicylic acid (ASA) first-time users and patients with ASA as maintenance therapy.**

**
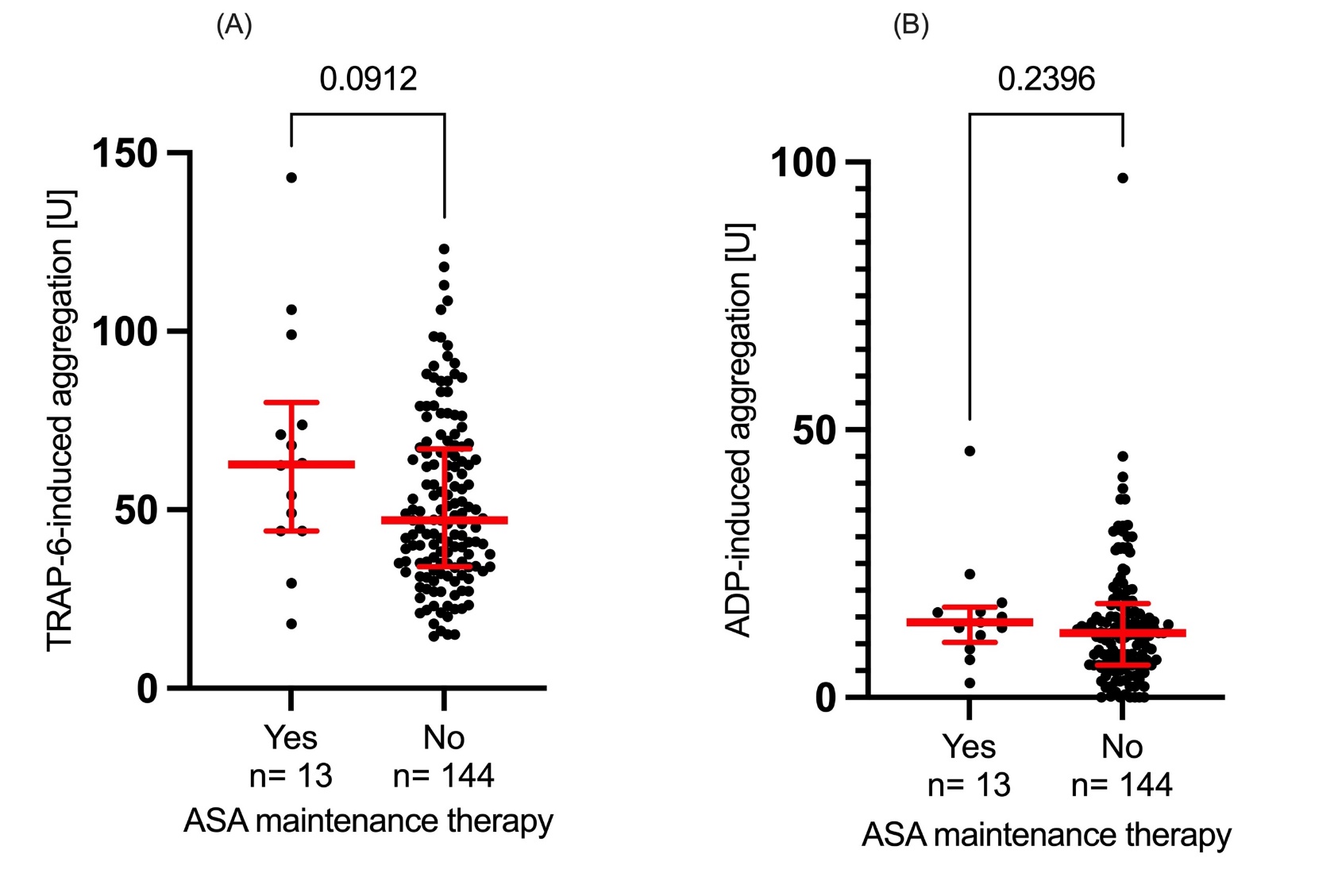
**

Red line shows median and interquartile range (IQR) for TRAP- and ADP- induced aggregation values measured for each of the groups.

## Figure S8. Distribution of ADP-induced aggregation according to the time to measurement after clopidogrel loading in hours

**
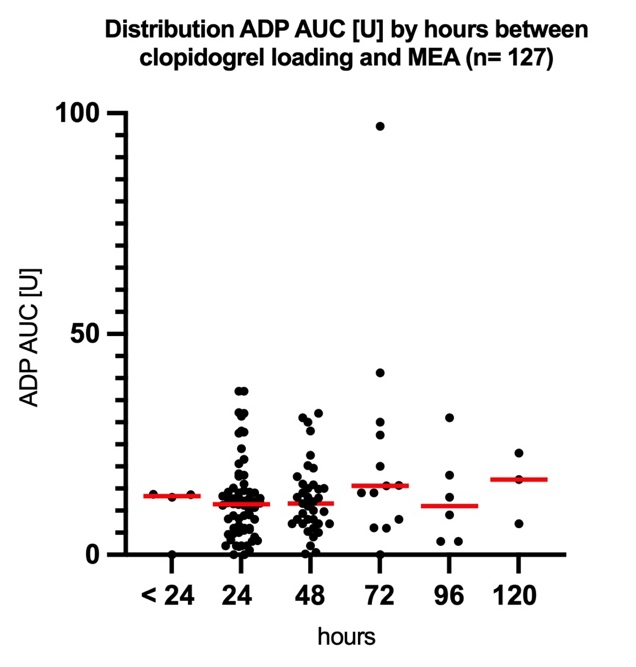
**

Red line shows median for ADP- induced aggregation values measured for each of the groups.

**Figure S9. (A) TRAP- and (B) ADP- induced aggregation according to the time of measurement after blood draw in minutes**

**
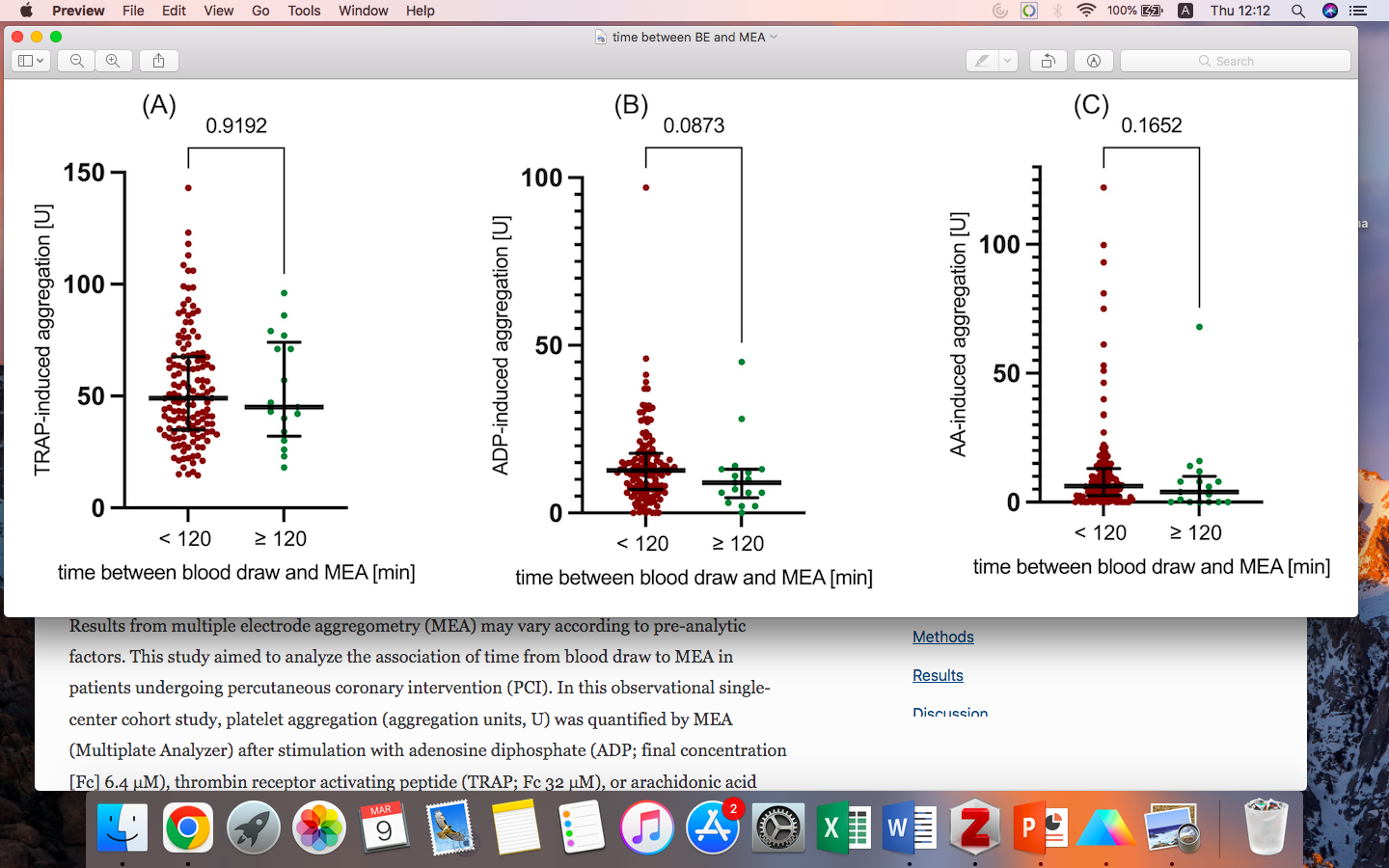
**

Black line shows median and interquartile range (IQR) for ADP- and TRAP- induced aggregation values measured for each of the groups.

**Figure S9. (A) TRAP-, (B) ADP- and (C) AA-induced aggregation values according to the center where the MEA measurement was performed**

**
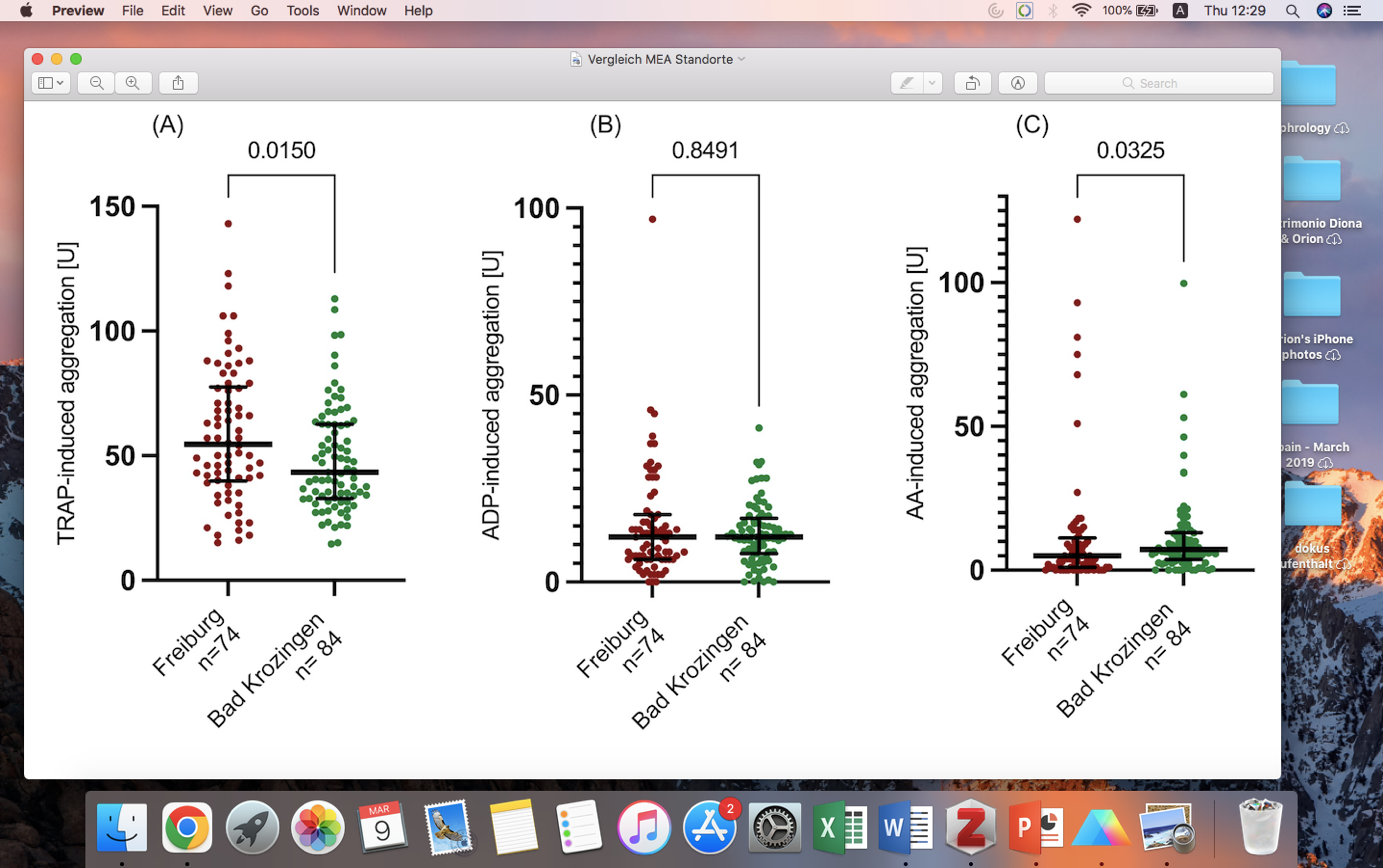
**Black line shows median and interquartile range (IQR) for ADP- and TRAP- induced aggregation values measured for each of the groups.
